# Is the glutamine story over?

**DOI:** 10.1186/s13054-016-1531-y

**Published:** 2016-11-10

**Authors:** Marie Smedberg, Jan Wernerman

**Affiliations:** 1 Department of Anesthesia and Intensive Care Medicine at Karolinska University Hospital Huddinge, Stockholm, Sweden; 2 Division of Anesthesia and Intensive Care Medicine at CLINTEC, Karolinska Instituetet, Stockholm, Sweden

**Keywords:** Hypoglutaminemia, Hyperglutaminemia, Critical care, Guidelines

## Abstract

Glutamine has been launched as a conditionally indispensible amino acid for the critically ill. Supplementation has been recommended in guidelines from international societies. Although data have been presented pointing out that glutamine supplementation may not be for everybody, recommendations for treatments and design of study protocols have included all critically ill patients. Results from more recent studies and meta-analyses indicate that indiscriminate use of glutamine supplementation in critically ill patients may actually cause harm rather than beneficial effects. This viewpoint sorts out arguments of controversy in the glutamine story.

## Glutamine is not for everybody, but possibly for some

The glutamine story is over in the sense that the hypothesis that all critically ill patients should be given extra glutamine supplementation has been demonstrated not to be valid [1–4]. However, the hypothesis that some critically ill patients may have a shortage of glutamine which needs to be corrected has not been tested appropriately [3, 4]. Originally a hypothesis of glutamine being conditionally essential in critical illness was launched [5, 6]. Although it was never proposed that this condition was a general feature of critical illness, the idea of glutamine supplementation was embraced for general use, not confined to patients with hypoglutaminemia only.

When glutamine supplementation studies are put together into meta-analyses, there is no longer a recommendation for general use in the critically ill. It is pointed out that studies involving patients on enteral nutrition and with an enterally administered supplementation are less likely to show beneficial effects from glutamine supplementation [7–10]. However, when separating studies employing glutamine supplementation by the parenteral route only, there is still one meta-analysis advocating the use of glutamine supplementation in this setting [9].

For many years it has been known that a low plasma glutamine concentration at ICU admission is associated with an unfavorable outcome [11, 12]. It is also demonstrated that hypoglutaminemia is not connected to mortality risk predictors such as APACHE II or SAPS. Hypoglutaminemia at ICU admission is actually an independent mortality predictor, adding mortality prediction accuracy to the APACHE II in consecutive unselected patients admitted to the ICU [12].

From existing data we know that hypoglutaminemia is not a particular feature of the sickest ICU patients [11, 12]. On the contrary, admission plasma glutamine is totally unrelated to mortality predictors such as APACHE II or SAPS III. Recruitment of only the very sickest patients for glutamine supplementation to counteract hypoglutaminemia is therefore not supported by the existing observational data.

The recruitment of ICU patients with severe sepsis and two or more organ failures to receive a very high dose of combined enteral and parenteral supplementation was therefore not a good idea [13]. Although there may be a selection bias in the recruitment [14, 15], the message from this study is very clear—there is no beneficial effect of indiscriminate glutamine supplementation, and there is perhaps harm [13]. There are three important remaining questions: (i) might harm be associated with administration of supraphysiologic doses of exogenous glutamine during hypocaloric nutrition; (ii) if so, what is the underlying mechanism; and (iii) might there be a subgroup of patients who benefit from glutamine supplementation?

It is of course important to point out that the statistical connection between hypoglutaminemia and an unfavorable outcome fulfills the criteria for a biomarker, but provides no proof of a causal connection. Launching the hypothesis that exogenous supplementation to achieve normoglutaminemia and thereby improve outcomes is not far-fetched, but this hypothesis is still to be proven. In all studies with a beneficial effect, the absence of a statistical connection between a change in plasma glutamine concentration and the advantage achieved is a clear limitation. If treatment of hypoglutaminemia should be the primary target for glutamine supplementation, the connection between plasma concentration and beneficial effects must be much better described.

Alongside the observational data associating hypoglutaminemia at ICU admission with unfavorable outcome, there is massive evidence that low abundance of glutamine in experimental systems and in animals is associated with low performance, in particular of the immune system and of the intestinal mucosa [16, 17]. Some of the reported effects are responses to glutamine supplementation outside the physiologic range, but on the contrary there were no reports of harmful or toxic effects at supraphysiologic levels. The idea to provide glutamine supplementation to all patients regardless of documented hypoglutaminemia was therefore embraced by a large number of investigators. The motivation behind not including glutamine plasma levels was often the difficulty in obtaining emergency plasma glutamine concentration determinations, and the perhaps erroneous idea that extra glutamine was not toxic.

## Plasma glutamine as proxy

**Fig. 1:**
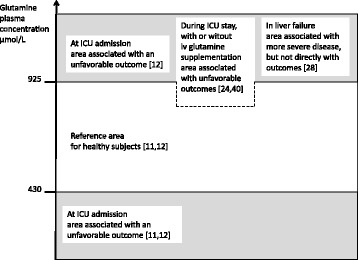
Updated summary of glutamine plasma concentration as a biomarker. *iv* intravenous

Can the plasma glutamine concentration guide us (Fig. 1)? In general we know that there is a poor correlation between plasma concentration and tissue concentrations [18–21]. Following elective surgery of moderate size there is a drop in muscle but not in plasma [19]. In critically ill subjects, on the contrary, there is a profound drop in muscle while the drop in plasma is variable, and always less dramatic [18, 20]. In other tissues like the intestinal mucosa, the tissue concentration and plasma concentration are more or less parallel during critical illness, and the gradient does not change as it does in muscle [22]. The glutamine plasma concentration is therefore not a perfect reflection of total glutamine availability; it is a low fraction of the total free glutamine pool, and the equilibration in the total free glutamine pool is very slow [23].

The relation between hypoglutaminemia and an unfavorable outcome is confined to the admission value of plasma glutamine [12, 24]. Beyond this, knowledge is scattered. The glutamine concentration at admission to the ward after an ICU stay > 96 hours does not predict outcome [24]. Among extreme ICU longstayers, a high plasma glutamine concentration is associated with poor outcome [25], but general hyperaminoaciemia and high concentrations of other amino acids were even stronger indicators of a poor outcome, so this particular observation was not specific for glutamine. In the study cited, the plasma concentration during ongoing IV glutamine supplementation was in the reference range, but values in the higher part of normal glutamine concentration interval added mortality prediction to the admission SAPS III [24]. Another report finds a high plasma glutamine level on day 8 of an ICU stay to correlate with 6-month mortality, regardless of whether enteral glutamine supplementation was given or not and regardless of admission mortality prediction [26]. In summary, there is therefore little evidence that the plasma glutamine concentration during ICU stay and post ICU stay can serve as an outcome predictor. Hyperglutaminemia is possibly associated with a poor outcome, which may be a part of general hyperaminoacidemia.

The low muscle free glutamine concentration in critical illness is probably a general phenomenon, although there are no reports of muscle glutamine in consecutive admissions [18]. This is in contrast to hypoglutaminemia in the critically ill, which occurs in only 1/3 of ICU admissions, and is not related to admission mortality prediction scores, although it adds predictive value to such scores [12].

The only patient group reported with hyperglutaminemia is those with acute liver failure [27]. Furthermore, in a study of consecutive patients admitted to the ICU, an association between hyperglutaminemia at ICU admittance and an unfavorable outcome was seen [12]. On the contrary, no general connection between hyperglutaminemia at ICU admission and an unfavorable outcome could be demonstrated in a selected population of only patients with liver failure [28].

Recently a high plasma glutamine concentration during critical illness has been suggested to be an ominous prognostic sign [2]. These data are less consistent compared with the data behind the original hypothesis involving hypoglutaminemia at ICU admittance. It has been clearly demonstrated that the plasma glutamine concentration in ICU survivors 24 h after ICU discharge is most often in the normal range and has no predictive value for outcomes [24]. In the same study it was observed that the plasma glutamine concentration during ongoing IV glutamine supplementation on the last day of the ICU stay was in the normal range, but was statistically associated with post-ICU mortality [24]. This association also remained when the ICU discharge SOFA score was used as an outcomes predictor. A post-hoc finding when ICU patients were randomized to a glutamine-containing enteral product was that an increase in plasma glutamine from ICU admission to day 8 of the ICU stay was associated with 6-month mortality [1]. Taken together these reports offer no clear picture, but call for a more systematic exploration of the relation between plasma glutamine concentration and outcomes during critical illness, with and without exogenous supplementation.

What is the substance behind the hypothesis that hypoglutaminemia is an indicator of glutamine shortage and therefore an indication for substitution. Is hypoglutaminemia an indication of low free glutamine levels in tissues? In critical illness this is true for muscle, but not for intestinal mucosa, although the reverse (low tissue means low plasma) is not necessarily true [22]. Is hypoglutaminemia an indicator of low peripheral export of glutamine to the splanchnic organs? In the very few publications on this issue, there is no obvious connection [29]. Is hypoglutaminemia an indicator of low de-novo glutamine production? Again, very limited information, but so far no connection has been observed between muscle glutamine synthase activity or free glutamine Ra and low plasma glutamine [30]. In summary, there is no obvious connection between hypoglutaminemia at ICU admission and signs of glutamine shortage, which does not rule out glutamine supplementation as a good idea but does not supply the hypothesis with a rational argument.

## Possible underlying mechanisms of harm and effect

Supplementation of glutamine may be administered by IV or enteral route, which is a both confusing and controversial topic. When identical doses of glutamine or a glutamine-containing dipeptide are given, IV administration produces a much higher plasma concentration compared with enteral administration [31, 32]. The distribution of a given dose of supplementation will therefore obviously differ in relation to the route of administration. This is accounted for in several meta-analyses, which present subgroup analyses according to the route of administration [7–10]. Unfortunately, to add to the confusion, the route for general administration of nutrition is not distinctively separated from the route for glutamine administration. Commonly in critically ill patients, nutrition is administered as a combination of enteral and parenteral supply. Enteral nutrition was for a long time considered superior to parenteral nutrition in terms of morbidity and mortality outcomes. Unfortunately most (if not all) of the studies behind this finding were biased in terms of patient selection. When the two routes of administration were prospectively compared in patients eligible for both routes, there were no differences in outcomes [33].

Studies with a parenteral administration of glutamine (or glutamine-containing dipeptides) are in general exhibiting more beneficial results compared with studies with enteral administration [7, 9]. The number of studies with a combined enteral and parenteral supplementation is low [13]. Is it reasonable to separate studies according to the route of glutamine administration? If the target is to achieve an effect upon plasma glutamine concentration—yes? If the target is to supply a certain amount of glutamine—not self-evident. As already indicated, the metabolic faith of administered glutamine will be different according to the route of administration. The question is whether this difference might be translated into a difference in clinical outcomes.

It is known that the export of glutamine from muscle tissue during critical illness is enhanced [29]. This export is a reflection of the development of sarcopenia, well known particularly in ICU longstayers [34, 35]. In a small longitudinal study the volume of this export did not change over time during the ICU stay [29]. This may be interpreted as the glutamine export from muscle being insufficient and therefore being a factor adding to the need for a prolonged ICU stay. An alternative interpretation may be that the longstayers with a maintained glutamine export actually were the selected survivors. Again, this calls for future studies to better understand glutamine kinetics during critical illness.

In an effort to study the mechanism behind plasma glutamine concentration and glutamine availability, the endogenous production of glutamine was quantified in terms of glutamine rate of appearance, a technique that employs isotopic labeled glutamine [30]. In a limited number of ICU patients there was no clear relationship between the plasma concentration of glutamine and the rate of appearance [36]. In addition, adding exogenous IV glutamine supplementation actually increased the rate of appearance. In summary, efforts to quantify glutamine availability by measurement of the rate of appearance will add information of glutamine kinetics during critical illness and may add information concerning the mechanisms behind beneficial effects as well as harm of glutamine supplementation.

A possible harmful effect is also reported in studies where extra glutamine supplementation is combined with supplementation of other additives, such as arginine and omega-3 fatty acids [26]. These studies also raise concern over extra glutamine supplementation, but studies with several additives in parallel are most often not conclusive. In general, studies combining several active interventions are almost universally adding confusion rather than new evidence.

## Are further studies over glutamine supplementation motivated?

So what is agreed upon? Indiscriminate use of extra glutamine supplementation for all critically ill patients is no longer on the agenda. On the contrary, nobody is advocating a glutamine-free diet for critically ill patients—but that is probably the end of agreement. All available enteral products for ICU nutrition contain glutamine, being a natural constituent of proteins, usually 7–8 % of the amino acid content. Simultaneously, conventional nutritional products for parenteral use do not contain glutamine due to glutamine instability in aqueous solution. Should the ideal parenteral product mimic the enteral? This question is further complicated by the fact that not all commercial sets of nutrition products contain the option of a choice between glutamine-free and glutamine-containing parenteral alternatives (on the same level as in enteral products).

An adequate evaluation of the glutamine hypothesis would be to supply patients with hypoglutaminemia at ICU admission with a dose of glutamine that normalizes plasma glutamine together with an adequate caloric and protein supply, not less than 50 % of energy expenditure and not less than 50 % of ESPEN-recommended protein supply. Such a protocol would necessitate the use of IV exogenous glutamine supplementation, because the effect of enteral supplementation upon plasma concentration is less predictable [26, 32, 37]. In addition, the supplementation of glutamine is not likely to be effective without being a part of optimal nutrition. Because this is a highly controversial issue today, the design of the study must consider the glutamine level, the caloric and protein intakes and the timing.

The absence of any close relationship between plasma glutamine concentration and the global glutamine status of the individual patient remains problematic. The connection between hypoglutaminemia at admission and unfavorable outcome still points to this group of critically ill patients as the most suitable candidates for supplementation.

The strongest argument for a prospective study is perhaps the absence of any mechanistic explanation of the harmful results, which is translated into a limited external validity of these results [38]. This should be compared with the results reported when critically ill patients are fed according to estimated caloric needs; results are then reported as beneficial or as no effect without harm [39]. Still, a key question is whether the plasma glutamine concentration might serve as a proxy endpoint between exogenous supplementation and an outcome-related endpoint. Such a proxy endpoint might allow titration of the supply to individualized dosing. Point-of-care instruments may be helpful when using such a proxy endpoint [40].

Is there any agreement over the need to further explore the subgroup of critically ill patients with hypoglutaminemia at ICU admission? This issue comes down to whether hypoglutaminemia is a biomarker reflecting a general shortage of physiologic reserve not fully covered by the conventional risk scoring systems, or whether it is a biomarker also reflecting a mechanistic role for glutamine. Although everybody would agree that there is insufficient solid knowledge, opinions remain apart.

## Conclusion

The concept of glutamine as a conditionally indispensible amino acid is dead, as is the concept of supplementing all critically ill patients. The hypothesis that patients with hypoglutaminemia at ICU admittance may benefit from IV supplementation to normalize the plasma glutamine levels has never been tested. Opinions deviate as to whether or not this hypothesis is worthwhile to explore.

## Abbreviations

APACHE: Acute Physiology And Chronic Health Evaluation
ESPEN: European Society of Parenteral and Enteral Nutrition
ICU: Intensive care unit
IV: Intravenous
SAPS: Simplified Acute Physiology Score
SOFA: Sequential Organ Failure Assessment
